# Safety and Effectiveness of Liv.52 DS in Patients With Varied Hepatic Disorders: An Open-Label, Multi-centre, Phase IV Study

**DOI:** 10.7759/cureus.60898

**Published:** 2024-05-23

**Authors:** Sachin K Shivnitwar, Ishwar Gilada, Atul V Rajkondawar, Sandeep K Ojha, Sandeep Katiyar, Navneet Arya, U. V Babu, Rajesh Kumawat

**Affiliations:** 1 General Medicine, D. Y. Patil Medical College, Pune, IND; 2 Skin and Sexually Transmitted Diseases, Unison Medicare and Research Centre, Mumbai, IND; 3 Internal Medicine, Government Medical College, Nagpur, Nagpur, IND; 4 Hyperbaric Medicine and Woundcare, Jayyush Hospital, Ahmedabad, IND; 5 Pulmonology Department, Apollo Spectra Hospital, Kanpur, IND; 6 Ayurveda, Sri Sai Institute of Ayurvedic Research and Medicine, Bhopal, IND; 7 Research and Development, Himalaya Wellness Company, Bengaluru, IND; 8 Medical Services and Clinical Development, Himalaya Wellness Company, Bengaluru, IND

**Keywords:** liv.52, hepatoprotective, hepatitis, drug-induced hepatotoxicity (dih), non-alcoholic fatty liver disease (nafld), alcoholic liver disease (ald)

## Abstract

Background

The hepatoprotective function of polyherbal formulation Liv.52 in chronic liver diseases is well recognized in published literature. The objective of this open-label, phase IV study was to further strengthen and validate its safety and effectiveness using a large patient pool in a real-world scenario and provide scientific data on symptomatic improvement and supportive treatment in liver function with improvement in quality of life.

Methods

Adult patients of either sex with one or more clinical symptoms like fatigue, nausea, anorexia, abdominal pain or discomfort, muscle cramps, jaundice, or any other signs and symptoms with a history suggestive of mild-to-moderate hepatic disorders like alcoholic liver disease (ALD), non-alcoholic fatty liver disease (NAFLD), drug-induced hepatotoxicity, or hepatitis were treated with two Liv.52 DS tablets (oral) twice daily for 12 weeks.

Results

Out of the 1000 enrolled patients, 962 (96%) completed the study with the following subgroups ALD: 375 (38.9%), NAFLD: 379 (39.3%), drug-induced hepatotoxicity: 78 (8.1%), hepatitis: 130 (13.5%). The mean age of enrolled patients was 37.7 years, and the majority of them, 785 (78.5%) were men. The common adverse events observed (with >1.5% incidence) in the study were abdominal pain: 26 (2.6%) and headache: 17 (1.7%). Liv.52 showed statistically significant improvement (P<0.0001) in various clinical signs and symptoms in the majority of patients namely, fatigue: 357/723 (49%), anorexia: 485/620 (78.2%), jaundice: 48/52 (92%). Majority of the patients showed significant improvements from baseline to end of 12 weeks in the liver function test parameters namely, aspartate aminotransferase: 633/840 (75.36%), alanine aminotransferase: 592/729 (81.21%), serum bilirubin: 244/347 (70.32%), alkaline phosphatase: 279/355 (78.59%) with P<0.0001 for all parameters. Statistically significant improvement (P<0.005) was also seen in all the components of the chronic liver disease questionnaire (CLDQ) scores from baseline to 12 weeks.

Conclusions

The study demonstrated that Liv.52 was hepatoprotective and well tolerated in the study population after treatment for 12 weeks. Significant improvements were seen in clinical signs and symptoms, laboratory parameters of liver function, and CLDQ scores from baseline to 12 weeks. No significant or new safety signals emerged from this study.

## Introduction

Chronic liver disease (CLD) affects >800 million individuals globally, leading to over two million deaths each year, accounting for 4% of all deaths worldwide [1]. Liver disease ranks as the 11th most prominent factor contributing to mortality and the 15th leading cause of disability-adjusted life-years. The highest impact is on patients aged between 25 to 49 years [2]. Overall liver disease leads to two million worldwide deaths, of which 18.3% are from India [3].

The progression of CLD starts with inflammation, followed by fibrosis with impaired normal functioning. The final stage is cirrhosis or scarring of the liver tissues, which is associated with a significant compromise of liver function [4]. Based on the etiology and pathogenesis, CLDs are classified into different types; the most common being alcoholic liver disease (ALD), non-alcoholic fatty liver disease (NAFLD) or metabolic-associated fatty liver disease (MAFLD), drug-induced hepatotoxicity (DIH), and chronic viral hepatitis [5]. Pharmacological interventions to manage CLD may include antiviral drugs (viral hepatitis), corticosteroids, and immunosuppressive agents (autoimmune hepatitis) apart from symptomatic management [4]. However, long-term use of antiviral medications has been associated with hepatotoxicity and antiviral resistance [5,6], while long-term use of immunosuppressants increases the risk of infections and causes metabolic disturbances [4,7]. This necessitates the identification and development of alternative medicines that are hepatoprotective in function and are less toxic or safe for long-term use.

Liv.52 is a polyherbal licensed ayurvedic medicine from the traditional system of medicine. Liv.52 is shown to be hepatoprotective, antioxidant, antiviral, and anti-inflammatory in function. As per the available information, it is indicated in the symptomatic improvement and supportive treatment of mild-to-moderate ALD, viral hepatitis, NAFLD, and DIH [7]. Liv.52 might be used as an adjuvant to other medications known for causing DIH (anti-tubercular drugs, antiretrovirals, chemotherapeutic agents, statins) [6-8]. Although the hepatoprotective function of Liv.52 in liver diseases is well recognized, the objective of this study was to further strengthen and validate its safety and effectiveness on a larger patient pool in a real-world scenario in scattered geography. It was aimed to generate clinical data related to symptomatic improvement, impact on quality of life (QOL), liver enzymes, and to assess the safety with higher doses (Liv.52 DS, two tablets, twice daily) when used in varied hepatic disorders.

## Materials and methods

Study design and ethical considerations

This open-label, single-arm, phase IV study was conducted from August 2022 to July 2023 at 37 sites across eight cities in India. The study protocol (CTRI registration number: CTRI/2022/08/044545) was reviewed and approved by the independent ethics committees at all participating sites (details of which have been provided in Appendices Table 4).The study was conducted in full compliance with ethical principles laid down by the World Medical Association -Declaration of Helsinki (Oct 2013) for medical research involving human subjects, Guidelines of the International Conference on Harmonization of Technical Requirements for Registration of Pharmaceuticals for Human Use (ICH) - Good Clinical Practices (GCP), ‘Ethical-Guidelines for Biomedical Research on Human Subjects’ issued by Indian Council of Medical Research (2017) and the applicable regulatory requirement in India (Schedule ‘Y’) and Central Council for Research in Ayurvedic Sciences. All patients provided written informed consent before participation in the study. As this was a Phase IV study, there was no formal sample size calculation planned. To meet the objective of validating the safety and effectiveness of the test product using a large patient pool in a real-world scenario a sizeable number of 1000 patients were planned to be enrolled in the study.

Study population

Patients (>18 years old) of either sex with one or more clinical symptoms like fatigue, nausea, anorexia, abdominal pain or discomfort, muscle cramps, jaundice, or any other symptoms with a history suggestive of mild-to-moderate hepatic disorder like ALD, which is characterized with a history of persistent alcohol intake and with liver function tests (LFT) suggestive of liver disease, NAFLD (patients with a history suggestive of impaired metabolic function and the final diagnosis was made through exclusion), DIH (patients with the specific history of drug intake attributed to causing liver injury like anti-tubercular, anti-retroviral therapies etc.) or hepatitis (patients with elevated LFT suggestive of hepatitis) were enrolled in the study as per protocol defined eligibility criteria. Patients of child-bearing potential were enrolled only if they agreed to use adequate and validated contraception during the study period. Patients suffering from severe hepatic conditions or complications like hepatocellular carcinoma, chronic hepatitis (hepatitis B or hepatitis C), portal hypertension, splenomegaly, hepatic encephalopathy, confusion, swelling/bleeding from veins, fluid accumulation in the abdomen, non-alcoholic steatohepatitis with cirrhosis as well as those undergoing active treatment for alcohol withdrawal syndrome, or those with ongoing treatment for CLD were excluded from the study. Patients with normal LFT or those with highly elevated LFT defined by alanine aminotransferase (ALT) and aspartate aminotransferase (AST) >5 times the upper limit for normal (ULN) or those with compromised hematological parameters (total white blood cells <3,000 cells/mm3, absolute neutrophils <1,500 cells/mm3, platelets <1,00,000/mm3) or patients with serum creatinine >2 mg/dL or creatinine clearance <60 mL/min (based on Modification of Diet in Renal Disease (MDRD)) at screening, were also excluded from the study. The study also excluded pregnant or lactating women and patients where prior treatment of Liv.52 DS was found to be ineffective or non-tolerable.

Study treatment

Eligible patients were treated with Liv.52 DS two tablets (oral) twice daily for 12 weeks or end of study (EOS).

Study assessments

Safety evaluations included monitoring of adverse events (AEs) throughout the study. All AEs were coded using Medical Dictionary for Regulatory Activities (MedDRA, Version 26.0). Clinical laboratory data for hematological and biochemical parameters, physical examinations, and vital signs were also evaluated. Efficacy evaluation included changes in LFT parameters (ALT, AST, bilirubin, and alkaline phosphatase (ALP) from baseline to EOS. The study also evaluated the changes in clinical signs and symptoms (fatigue, nausea, anorexia, abdominal pain or discomfort, muscle cramps, jaundice, and any other symptoms related to mild-moderate hepatic disorder) for all patients. Patient-reported QOL assessments were performed using the chronic liver disease questionnaire (CLDQ, 29 items in the following domains: abdominal symptoms, fatigue, systemic symptoms, activity, emotional function, and worry). All CLDQ responses were rated from one (denoting “all of the time”) to seven (denoting “none of the time”) for all patients.

Statistical analysis

Safety was analyzed for the safety analysis set (all patients who were allocated to treatment and received at least one dose of the study drug) and was summarized descriptively. Efficacy parameters were analyzed using the per-protocol (PP) analysis set (all patients who completed the study as per protocol). Continuous data were analyzed by paired t-test or Wilcoxon signed-rank test, wherever appropriate, for comparing pre- and post-treatment data. Categorical data like abnormal to normal shift and overall improvement were analyzed using McNemar's test and Z-test. All statistical tests and confidence intervals are two-sided unless otherwise stated, P<0.05 was considered significant.

All statistical analyses were performed using SAS® software (version 9.4, SAS Institute, Cary, USA).

## Results

Demographic characteristics

**Figure 1:**
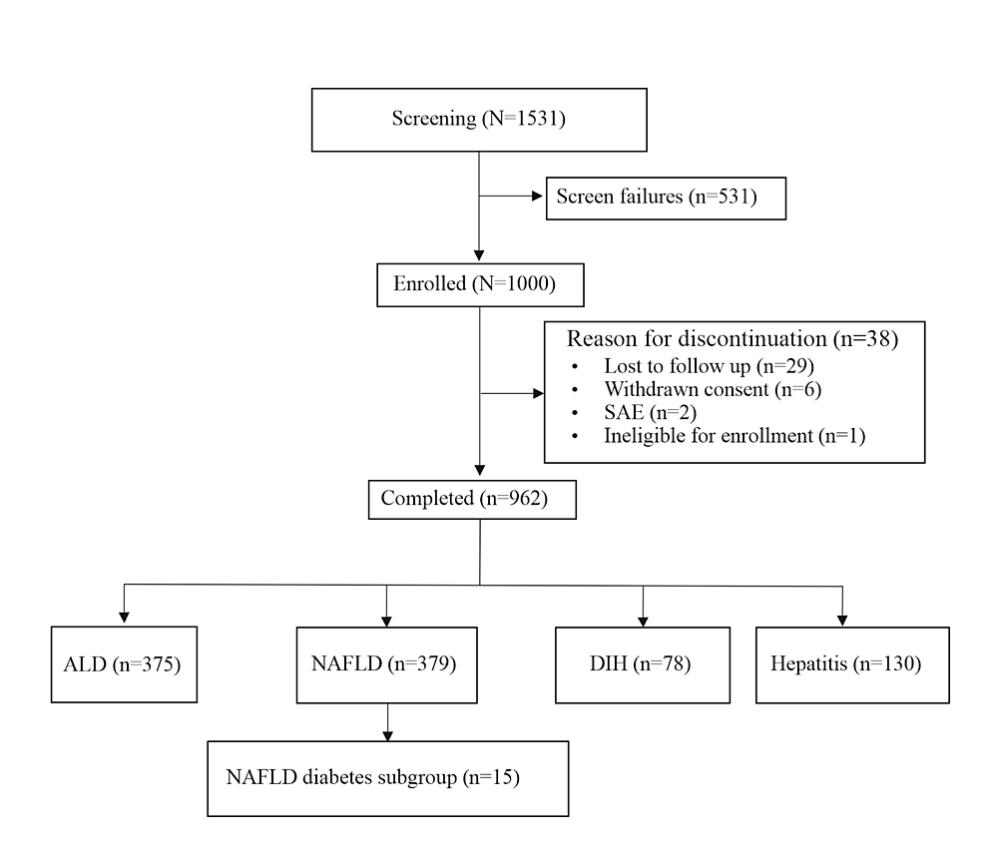
Patient Disposition (CONSORT Diagram) ALD: Alcoholic liver disease; DIH: Drug-induced hepatotoxicity; NAFLD: Non-alcoholic fatty liver disease; SAE: Serious adverse event; CONSORT: Consolidated Standards of Reporting Trials.

Adequate number of subjects were required to be screened to achieve the enrolment of 1000 subjects as per eligibility criteria. Overall, a total of 1,531 patients were required to be screened for eligibility to enrol 1,000 patients who were comprised of ALD: 387 (38.7%), NAFLD: 391 (39.1%), DIH: 85 (8.5%), hepatitis: 137 (13.7%) as per defined criteria. Most of the enrolled patients were nondiabetic 955 (95.5%) however, the remaining 45 (4.5%) patients did have diabetes. Of these, 962 (96%) patients completed the study (Figure 1).

The mean age of the enrolled patients was 37.7 years; the majority were men: 785 (78.50%) and all patients were of Asian origin (Table 1).

**Table 1:** Demographic Characteristics of Patients (All Enrolled Patients) BMI: Body mass index; SD: Standard deviation.

Demographics	Patients (N=1,000)
Age (years): Mean (SD)	37.7 (10.10)
Male, n (%)	785 (78.50)
Weight (kg): Mean (SD)	65.0 (11.02)
Height (cm): Mean (SD)	164.8 (8.10)
BMI (kg/m^2^): Mean (SD)	23.8 (3.59)
Asian, n (%)	1,000 (100)

Safety

Overall, 215 (21.5%) unique patients reported a total of 286 AEs, of which most AEs: 245 (85.6%) were of mild severity. Severity was assessed using Common Terminology Criteria for Adverse Events (CTCAE, Version 5.0). Causality assessment of these adverse events to confirm the relationship to the study drug was performed through the World Health Organization - Uppsala Monitoring Centre (WHO-UMC) causality assessment criteria. The treating investigators assessed that most of the AEs, 283 (98.9%) were not related to the study drug. Only three patients experienced three AEs, which were found to be related to study drug or adverse drug reactions mainly mild abdominal pain: 2 (0.2%) and moderate acidity: 1 (0.1%). Two serious adverse events (SAEs) were also reported during the study (death - one case of road traffic accident, and one case of worsening of tuberculosis). However, none of the SAEs were found to be related to the study treatment.

The most common (>1.5% incidence) AEs reported were abdominal pain: n=26 (2.6%) and headache: n=17 (1.7%) and both were deemed to be not related to the study treatment. Other AEs indicating lack of efficacy (maybe due to disease progression, lifestyle changes, or other co-morbidities) reported were elevations in the following parameters: ALP 53 (5.3%), bilirubin 35 (3.5%), AST 30 (3%), and ALT 27 (2.7%) (Table 2). There were no significant changes or worsening in hematological parameters, renal parameters (creatinine), or vital signs during the study.

**Table 2:** Summary of Adverse Events ADRs: Adverse drug reactions; AE: Adverse events; SAEs: Serious adverse events.

Adverse Event	Number of subjects: n (%)	Mild	Moderate	Severe
Blood alkaline phosphatase increased	53 (5.3%)	46	6	1
Blood bilirubin increased	35 (3.5%)	28	5	2
Aspartate aminotransferase increased	30 (3%)	26	2	2
Alanine aminotransferase increased	27 (2.7%)	23	2	2
Abdominal pain	26 (2.6%)	26	-	-
Headache	17 (1.7%)	17	-	-
Vomiting	13 (1.3%)	13	-	-
Pyrexia	13 (1.3%)	11	2	-
Hemoglobin decreased	12 (1.2%)	-	12	-
Cough	7 (0.7%)	7	-	-
Gastric irritation	7 (0.7%)	6	1	-
Nasopharyngitis	7 (0.7%)	7	-	-
Back pain	6 (0.6%)	6	-	-
Pain	5 (0.5%)	5	-	-
Rhinorrhea	3 (0.3%)	3	-	-
Fatigue	3 (0.3%)	3	-	-
Diarrhea	3 (0.3%)	3	-	-
Liver function tests increased	2 (0.2%)	1	1	-
Hepatomegaly	2 (0.2%)	2	-	-
Pruritus	2 (0.2%)	2	-	-
Abdominal distension	1 (0.1%)	1	-	-
Arthralgia	1 (0.1%)	1	-	-
Decreased appetite	1 (0.1%)	1	-	-
Dyspepsia	1 (0.1%)	1	-	-
Genital ulceration	1 (0.1%)	1	-	-
Insomnia	1 (0.1%)	1	-	-
Jaundice	1 (0.1%)	1	-	-
Nausea	1 (0.1%)	1	-	-
Somnolence	1 (0.1%)	1	-	-
Urinary tract infection	1 (0.1%)	1	-	-
White blood cell count decreased	1 (0.1%)	1	-	-
Total	284	245	32	7
Number of AEs (mild, moderate, severe)	284 (including 3 ADRs: 2 mild abdominal pain, 1 moderate acidity)			
Number of SAEs	2 (1 road traffic accident, 1 worsening of tuberculosis)			
Number of total adverse events (AEs, SAEs)	286			

Liver function test (LFT): overall impact

**Figure 2:**
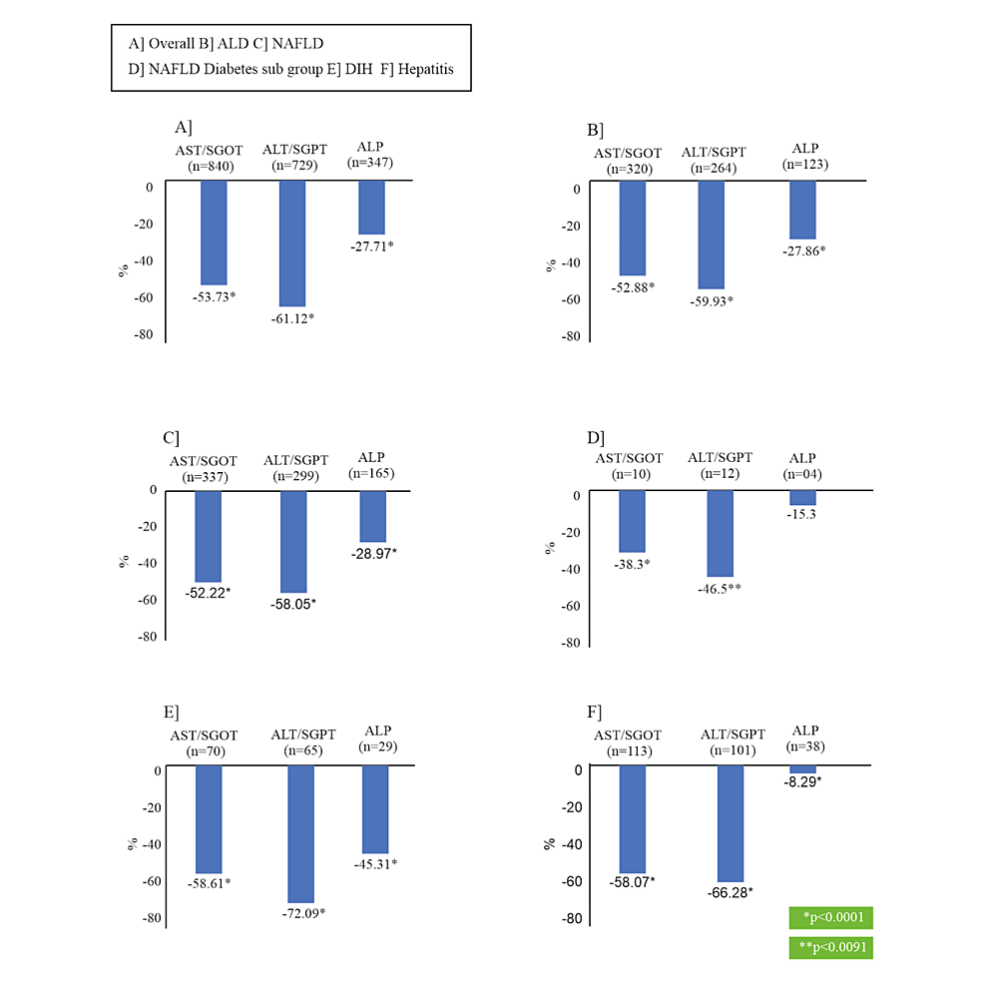
Average Percent Reduction in LFT Values for Patients, who had Abnormal Values at Baseline A] Overall Patient Population, B] Patients with ALD, C] Patients with NAFLD, D] Patients with NAFLD and Diabetes, E] Patients with DIH, F] Patients with Hepatitis ALD: Alcoholic liver disease; ALP: Alkaline phosphatase; ALT: Alanine aminotransferase; AST: Aspartate aminotransferase; DIH: Drug-induced hepatotoxicity; LFT: Liver function test; NAFLD: Non-alcoholic fatty liver disease; SGOT: Serum glutamic oxaloacetic transaminase; SGPT: Serum glutamic pyruvic transaminase.

**Figure 3:**
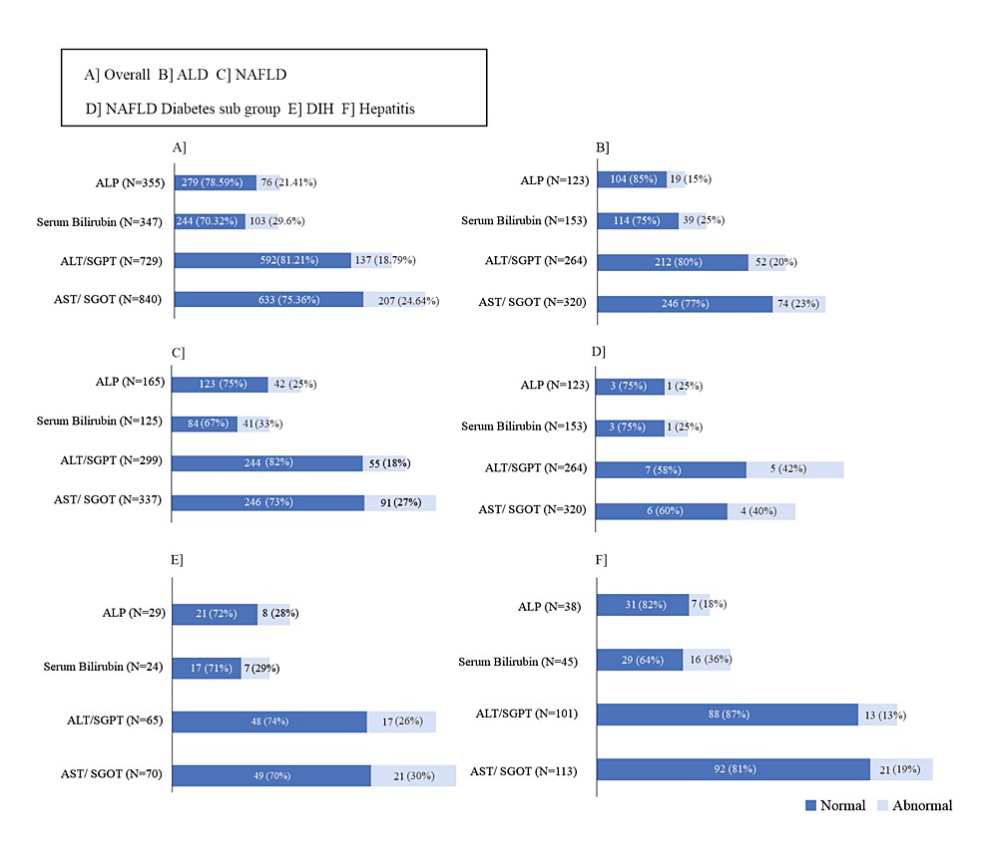
Patient-wise Shift (Number and Percentage) from Abnormal to Normal Values in LFT from Baseline to EOS (PP Analysis Set) A] Overall Patient Population, B] Patients with ALD, C] Patients with NAFLD, D] Patients with NAFLD and Diabetes, E] Patients with DIH, F] Patients with Hepatitis ALD: Alcoholic liver disease; ALT: Alanine aminotransferase; ALP: Alkaline phosphatase; AST: Aspartate aminotransferase; DIH: Drug-induced hepatotoxicity; LFT: Liver function test; NFALD: Non-alcoholic fatty liver disease; PP: Per protocol; SGOT: Serum glutamic oxaloacetic transaminase; SGPT: Serum glutamic pyruvic transaminase.

A significant improvement (P<0.0001) was observed in all LFT parameters (from baseline to EOS). The mean (SD) AST level reduced from 113.7 (98.20) U/L at baseline to 37.0 (39.30) U/L at 12 weeks in the study. Out of 840 patients who had elevated AST levels at baseline, 633 (75.36%) patients returned to normal at EOS. Likewise, the mean (SD) ALT levels also reduced significantly from 123.8 (89.01) U/L at baseline to 36.9 (53.64) U/L at EOS. Out of 729 patients who had elevated ALT levels at baseline, 592 (81.21%) patients returned to normal at EOS. Similarly, most of the patients, 244/347 (70.32%) showed improvements in their bilirubin levels and 279/355 (78.59%) in ALP levels from baseline to EOS. Similar patterns were observed in all subgroups of patients for all LFT parameters (Appendices Table 5 and Figures 2, 3). Figure 2 shows the average percent reduction in LFT values in patients, who had abnormal values at baseline, and Figure 3 shows the patient-wise shift of abnormal values of LFT parameters at baseline to normal LFT values at EOS.

LFT in patients with ALD

Overall, patients with abnormal AST (n=320), ALT (n=264), bilirubin (n=153), and ALP (n=123) values at baseline demonstrated a significant reduction of 52.88%, 59.93%, 26.01%, and 27.86%, respectively at EOS (all P<0.0001) (Figure 2B). Figure 3B shows that a majority of patients had a shift from abnormal to normal LFT parameters at EOS. Additionally, 81 out of 113 patients (71%) with ALD had their AST/ALT ratio shifted from >1.5 to ≤1.5 at EOS, and all 43 patients (100%) of ALD with elevated bilirubin levels (>3 mg/dL) at baseline had their levels returned to ≤ 3 mg/dL at EOS, thereby suggesting normalization of altered liver function in ALD [9].

LFT in patients with NAFLD

A total of 337 patients with abnormal AST, 299 patients with abnormal ALT, and 165 patients with abnormal ALP values at baseline had their AST, ALT, and ALP levels reduced by 52.22%, 58.05%, and 28.97% respectively, at EOS (all P<0.0001) (Figure 2C). Figure 3C indicates the overall majority of patients demonstrated a shift from abnormal to normal LFT parameters at EOS.

LFT in patients with NAFLD and diabetes

Ten patients with abnormal AST, 12 patients with abnormal ALT, 4 patients each with abnormal bilirubin and ALP levels at baseline demonstrated a 38.3%, 46.5%, 16.7%, and 15.3% reduction in AST (P<0.0277), ALT (P<0.0091), bilirubin, and ALP levels respectively at EOS (Figure 2D). The majority of patients in this subgroup showed a shift to normal LFT parameters at EOS (Figure 3D).

LFT in patients with DIH

A total of 70 patients with DIH and abnormal AST values at baseline had their AST levels reduced by 58.61% at EOS (P<0.0001). Similarly, 65 patients with abnormal ALT, 24 patients with abnormal bilirubin levels, and 29 patients with abnormal ALP levels at baseline demonstrated 72.09%, 47.38%, and 45.31% reduction in their ALT, bilirubin, and ALP levels respectively at EOS (all P<0.0001) (Figure 2E). Majority of the patients had a shift to normal LFT parameters at EOS (Figure 3E).

A subgroup analysis was also performed for patients with DIH due to anti-tubercular and anti-retroviral treatment. Sixteen patients with DIH due to anti-tuberculosis treatment reported a change in mean value from 202 U/L to 61 U/L or a reduction of 141 U/L (70%) in mean ALT levels from baseline within 2-3 weeks (P<0.0001), indicating the preliminary trend of the efficacy of the study drug within 2-3 weeks in this subgroup. Similarly, higher level of ALT and AST, in 15 patients with DIH due to retroviral therapy was also significantly reduced at 12 weeks.

LFT in patients with hepatitis

Overall, 113 patients with abnormal AST levels at baseline reported a 58.07% reduction in AST levels at EOS (P<0.0001). Similarly, 101 patients with abnormal ALT, 45 patients with abnormal bilirubin, and 38 patients with abnormal ALP levels at baseline reported 66.28%, 17.5%, and 8.29% reduction in their ALT, bilirubin, and ALP levels at EOS respectively (P<0.0001 for all) (Figure 2F). The number of patients who demonstrated a shift from abnormal LFT parameters to normal at EOS is presented in Figure 3F.

Impact on anemia of chronic disease (ACD)

Out of 213 patients with low hemoglobin (male <12 gm/dL, female <10 gm/dL) at baseline, 62 (29.1%) had their mean corpuscular volume (MCV), mean corpuscular hemoglobin (MCH), and mean corpuscular hemoglobin concentration (MCHC) values normal, which clinically indicates a probable case of ACD. In these 62 (29.1%) patients, statistically significant improvements in mean hemoglobin levels (baseline: 10.8 gm/dL; EOS: 12.54 gm/dL; P<0.0001) were observed. Although ACD cannot be confirmed solely based on blood count alone, these results show an encouraging trend, which can be explored further in iron profiling studies.

Clinical signs and symptoms

Overall, most of the patients with clinical symptoms at baseline became symptom-free at EOS. Amongst patients who had symptoms of fatigue/tiredness/weakness at baseline, 357 (49%) out of 723 were found to be symptom-free at EOS. Of patients with symptoms of nausea at baseline, 419 (79.3%) out of 528 became symptom-free at EOS. Among patients with abdominal pain symptoms and muscle cramps at baseline, 583 (76%) and 297 (71%) patients became symptom-free at EOS, respectively (Table 3).

**Table 3:** Change from Baseline to EOS in Clinical Signs and Symptoms (PP analysis set) ALD: Alcoholic liver disease; DIH: Drug-induced hepatotoxicity; EOS: End of study; NAFLD: Non-alcoholic fatty liver disease; PP: Per protocol.

Signs and symptoms (N)	No (%) of patients with signs and symptoms at baseline who remained with signs and symptoms at EOS	No (%) of patients with signs and symptoms at baseline who became symptom free at EOS
All patients		
Fatigue/Tiredness/Weakness (n=723)	366 (50.62)	357 (49.38)
Nausea (n=528)	109 (20.64)	419 (79.36)
Anorexia (n=620)	135 (21.77)	485 (78.23)
Abdominal pain/Abdominal discomfort (n=771)	188 (24.38)	583 (75.62)
Muscle cramp/Muscle Spasm (n=419)	122 (29.12)	297 (70.88)
Jaundice (n=52)	4 (7.69)	48 (92.30)
Patients with ALD		
Fatigue/Tiredness/Weakness (n=250)	118 (47.2)	132 (52.8)
Nausea (n=204)	36 (17.65)	168 (82.35)
Anorexia (n=207)	32 (15.46)	175 (84.54)
Abdominal pain/Abdominal discomfort (n=335)	93 (27.76)	242 (72.24)
Muscle cramp/Muscle Spasm (n=162)	34 (20.99)	128 (79.01)
Jaundice (n=17)	2 (11.76)	15 (88.24)
Patients with NAFLD		
Fatigue/Tiredness/Weakness (n=282)	133 (47.16)	149 (52.84)
Nausea (n=200)	56 (28)	144 (72)
Anorexia (n=270)	69 (25.56)	201 (74.44)
Abdominal pain/Abdominal discomfort (n=282)	51 (18.09)	231 (81.91)
Muscle cramp/Muscle Spasm (n=185)	80 (43.24)	105 (56.76)
Jaundice (n=10)	0	10 (100)
Patients with NAFLD and Diabetes		
Fatigue/Tiredness/Weakness (n=11)	3 (27.27)	8 (72.73)
Nausea (n=3)	0	3 (100)
Anorexia (n=7)	0	7 (100)
Abdominal pain/Abdominal discomfort (n=10)	1 (10)	9 (90)
Muscle cramp/Muscle Spasm (n=5)	1 (20)	4 (80)
Jaundice (n=2)	0	2 (100)
Patients with DIH		
Fatigue/Tiredness/Weakness (n=74)	58 (78.38)	16 (21.62)
Nausea (n=48)	11 (22.92)	37 (77.08)
Anorexia (n=45)	11 (24.44)	34 (75.56)
Abdominal pain/Abdominal discomfort (n=34)	11 (32.35)	23 (67.65)
Muscle cramp/Muscle Spasm (n=12)	3 (25)	9 (75)
Jaundice (n=2)	0	2 (100)
Patients with hepatitis		
Fatigue/Tiredness/Weakness (n=117)	57 (48.72)	60 (51.28)
Nausea (n=76)	6 (7.89)	70 (92.11)
Anorexia (n=98)	23 (23.47)	75 (76.53)
Abdominal pain/Abdominal discomfort (n=120)	33 (27.5)	87 (72.5)
Muscle cramp/Muscle Spasm (n=60)	5 (8.33)	55 (91.67)
Jaundice (n=23)	2 (8.7)	21 (91.3)

Improvements in anorexia from baseline to EOS

**Figure 4:**
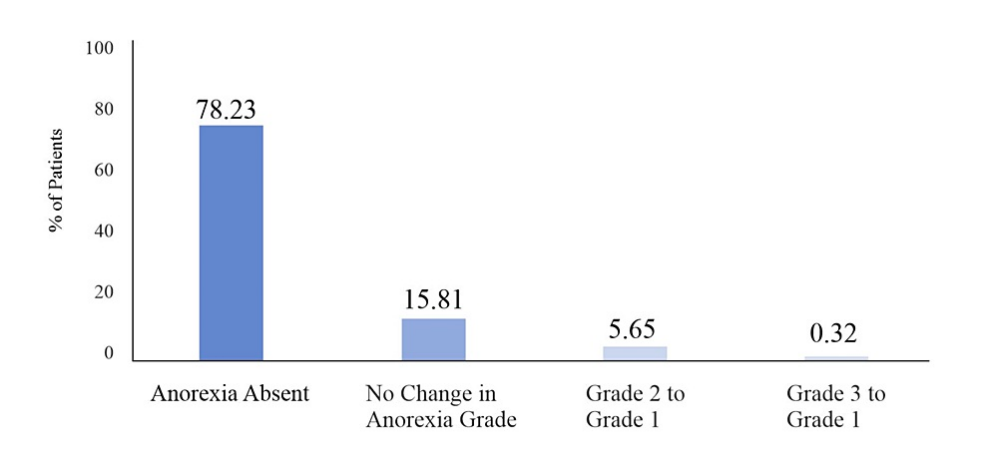
Impact on Anorexia at EOS (Per Protocol analysis set)

Overall, 620 (64.45%) out of 962 patients who completed the study, had anorexia at baseline (grade 1: 474, grade 2: 140, grade 3: six patients respectively). Of these, 485 (78.23%) patients were completely relieved of anorexia symptoms at EOS. Out of the remaining patients, about 35 (5.6%) showed grade reduction (Grade 2/3 to Grade 1) from baseline to EOS, while 98 (15.81%) showed no grade reduction for anorexia (Figure 4).

Quality of life assessment

**Figure 5:**
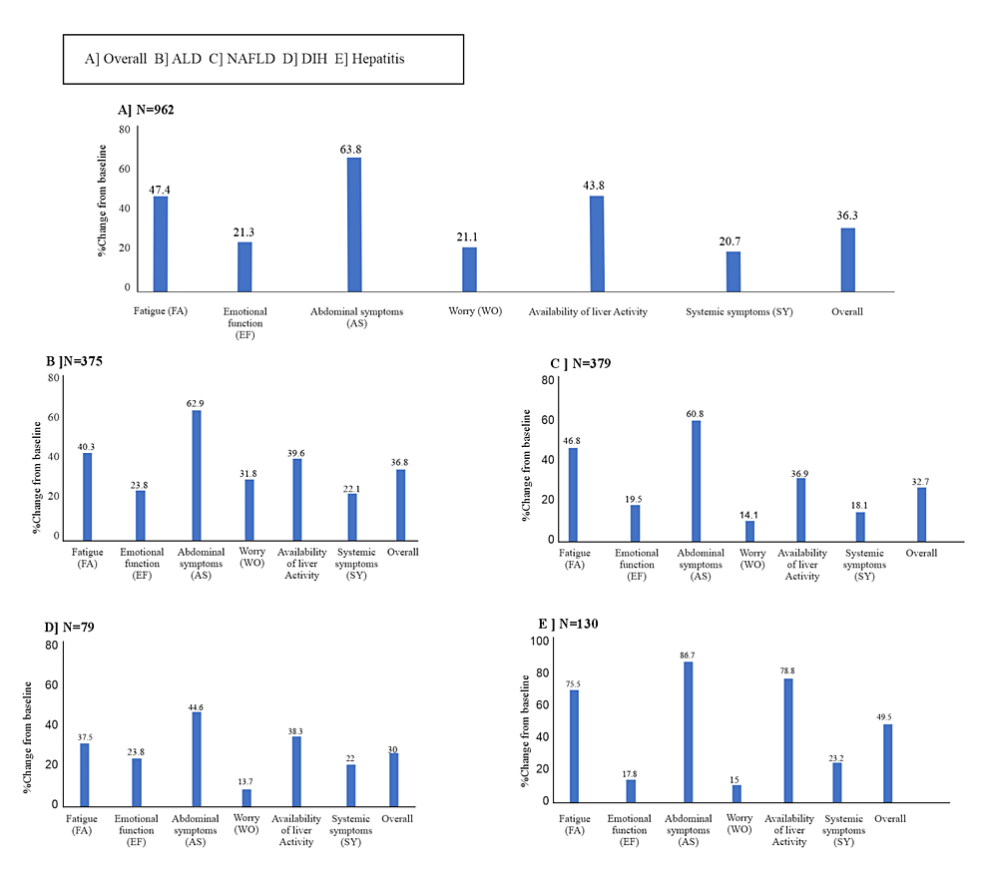
Improvements in CLDQ scores in Patients (PP Analysis Set) A] Overall Patient Population, B] Patients with ALD, C] Patients with NAFLD, D] Patients with DIH, E] Patients with Hepatitis ALD: Alcoholic liver disease; AS: Abdominal symptoms; CLDQ: Chronic Liver Disease Questionnaire; DIH: Drug-induced hepatotoxicity; EF: Emotional function; FA: Fatigue; NAFLD: Non-alcoholic fatty liver disease; PP: Per protocol; SY: Systemic symptoms; WO: Worry.

The mean CLDQ scores (overall and subdomains) improved significantly from baseline to EOS (Figure 5 and Appendices Table 6).

## Discussion

This open-label, phase-IV study demonstrated that Liv.52 was well tolerated and was hepatoprotective in patients with varied hepatic disorders in the study population. Statistically significant (P<0.0001) improvements were seen in clinical symptoms and liver enzyme parameters studied, from baseline to EOS, in the overall population as well as in all four subgroups of patients (ALD, NAFLD, DIH, hepatitis). Most of the patients in this study who had abnormal levels of liver enzymes at baseline became normal after the treatment with the study drug for 12 weeks. This statistical significance was translated into clinical significance, which was evident through improvement in signs, symptoms, and QOL of the participating patients.

Among 113 patients with ALD, 81 (71%) patients demonstrated a shift in their AST/ALT ratio from >1.5 to <1.5, and all 43 patients (100%) with significantly high bilirubin levels (>3 mg/dL) shifted to <3 mg/dL at EOS. The outcome of both the parameters in ALD, suggests normalization of altered liver function following the intervention of Liv.52, although this may be attributed to uncertainty (which is beyond control on outpatient studies) of alcohol consumption and abstinence in these patients [9]. Normalization of LFT in patients with DIH is usually observed following the discontinuation of ongoing treatments (anti-tuberculosis treatment anti-retroviral therapies or any other drug). However, in the current study, normalization could be achieved in patients, even after the continuation of their ongoing respective therapies. Additionally, improvement in LFT was also seen in the patients who had DIH due to other drugs like NSAIDS (etoricoxib), hypouricemic drug (febuxostat), DMARDs (hydroxychloroquine, sulfasalazine), and immunosuppressant (methotrexate). This is an important observation suggesting the hepatoprotective function of Liv.52 in such patients with DIH. However, more studies are warranted with a larger sample size to establish the hepatoprotective role of Liv.52 as an adjuvant with anti-tubercular or anti-retroviral therapies for the prevention and treatment of DIH.

Overall, our results were consistent with the earlier published studies demonstrating the hepatoprotective function of Liv.52 [8,10,11]. Liv.52 is known to decrease intrahepatic edema inhibiting necrosis; thereby relieving intrahepatic cholestasis and aiding the regeneration of hepatic cells [7]. It is also known to help in membrane stabilization by normalization of sodium-potassium-ATPase in the liver [11]. These activities directly or indirectly affect cellular and metabolic functions, including inhibition of oxidative DNA degradation, free radical scavenging, hydrogen peroxide inhibition, iron chelation, downregulation of tumor necrosis factor-α, replenishment of growth-stimulating hormone levels in the hepatocytes, and strong antiviral activity inhibiting viral attachment and penetration [12,13] and thus helps in maintaining the integrity of liver tissue and restoring its function [10].

Liv.52 is also known to effectively reverse the metabolic and histological changes associated with high-fat diet-induced NAFLD [14]. Consistent with this, in the current study, Liv.52 was effective in not just relieving the associated clinical signs and symptoms and reducing the LFT levels significantly in patients with NAFLD but was also found to be effective in abnormal to normal shift of liver parameters from baseline to EOS. NAFLD is strongly associated with obesity and type 2 diabetes mellitus (T2DM); it can be considered as the hepatic manifestation of the metabolic syndrome [15]. There is a 1.87-fold increased risk of cardiovascular events in NAFLD in the presence of T2DM and increased diabetes-related microvascular complications such as chronic kidney disease and retinopathy [16]. The present study noted a reduction in the clinical symptoms in patients of NAFLD with diabetes after treatment with Liv.52 for 12 weeks. However, considering the very low sample size, in this subgroup, larger studies are required to demonstrate the beneficial role of Liv.52 in this patient population.

A significant complication of CLD is malnutrition, due to poor dietary intake owing to anorexia. The current study found that out of 620 patients who had anorexia at baseline, 485 (78.23%) became symptom-free with respect to anorexia, following Liv.52 treatment.

Liver diseases can be exhausting for many patients due to their chronic nature. Assessing health-related QOL is important to understand the psychological, social, and functional aspects of the disease that directly impact everyday functioning [17]. Furthermore, it has been demonstrated that QOL exhibits substantial clinically significant associations with liver function parameters (albumin, bilirubin, ALP, and albumin-to-ALP ratio). Hence, inadequate management can adversely affect survival in these patients [17,18]. An improvement in liver parameters and the clinical signs and symptoms of liver diseases directly impacts the QOL of the patients. This correlation was evident in the current study, wherein improvements in LFT and clinical signs and symptoms were corroborated with improvements in CLDQ scores in all patients.

Safety of Liv.52 was further established as most AEs were mild in severity and unlikely related to the treatment. There was no treatment discontinuation due to AEs. Considering the large population size of this study, there were only three adverse drug reactions of mild-to-moderate severity. As per European Medical Association guidelines (summary of product characteristics, SmPC, 2009) such incidences of adverse drug reactions are termed as uncommon or infrequent. There were no treatment-emergent SAEs reported in the study. The safety information generated in this study is consistent with the known safety profile of Liv.52. No new safety concerns emerged from the study showing no concern about using Liv.52 DS two tablets twice daily for 12 weeks. No concern of nephrotoxicity (as per the assessment of serum creatinine) was observed in the study. In a small patient population, 145 (14.5%) lack of efficacy was observed (elevation of liver parameters), which may be attributed to disease progression, lifestyle changes, or other co-morbidities. There are approximately 40 commercial hepatoprotective polyherbal formulations that are available in the Indian market. However, evidence-based data can be found for only a few of these formulations [19]. Of these, Liv.52 has been widely studied for the therapy of CLD [20]. The outcome of the present study is in line with a phase IV study on liver care supplement ursodeoxycholic acid conducted in 235 patients with varied liver diseases (ALD, NAFLD, hepatitis) demonstrating a reduction in liver enzymes (ALT, AST) by 46%-55% in 12 weeks [21].

This registered real-world study is one of the first of its kind in the herbal segment, involving a large patient population with varied hepatic disorders with certain credibility in terms of standards and process of clinical research. The study was multicentric in nature and involved investigators from both allopathic and ayurvedic disciplines of medicine, to ensure variability and minimize the bias when the entire set of patient population is considered. The study used Oracle clinical remote data capture system which is 21 CFR part 11 compliant electronic data capture software, which ensures the credibility and integrity of the data. All AEs were coded using MedDRA, which further strengthens the assessments and reporting of safety in any study. The severity of adverse events was assessed through CTCAE, Version 5.0. All biochemical and hematological assessments were carried out at an accredited central laboratory to ensure uniformity, reliability, and accuracy of data.

While this phase IV study demonstrated the hepatoprotective effects and good tolerability of Liv.52 in patients with various hepatic disorders, the outcome should be interpreted carefully keeping in mind some notable limitations. One major limitation was the open-label design without placebo control, which could introduce bias in inference for the effectiveness of the product. Additionally, the diagnosis of the patients was made based on the available history and evidence at the investigator’s discretion. For example, NAFLD was not confirmed by fibro scan or biopsy in the patient population, which is the gold standard for diagnosis of such patients. The study also did not account for the potential confounding effects of diet, lifestyle, and alcohol abstinence (especially in the alcoholic liver disease group), which could have played an important role in managing such disorders. However, considering the nature of the study, these limitations are acceptable, making it difficult to draw definitive conclusions about Liv.52's effectiveness in the management of these patients. Therefore, the outcome should be considered as its possible role in symptomatic improvement and supportive treatment with a fairly acceptable safety profile of the product. Larger, sample size-driven controlled studies with strict diagnostic criteria and assessments as per recommended global therapeutic guidelines are warranted to further establish Liv.52's hepatoprotective role. Despite these limitations, the study provides real-world evidence supporting Liv.52's safety and potential benefits in varied hepatic disorders. 

## Conclusions

This large, multi-centre phase IV study provides real-world evidence that the polyherbal formulation of Liv.52 is an efficacious and safe option for patients with various hepatic disorders, including ALD, NAFLD, DIH, and viral hepatitis. Apart from limitations, which are inherited characteristics of such types of studies, some robust processes of clinical research were adopted in this study. Treatment with Liv.52 for 12 weeks led to significant improvement in clinical symptoms, liver enzyme levels, and health-related QOL across different patient subgroups. Despite many limitation factors, statistically significant improvement in LFT parameters along with favorable trends in terms of signs, symptoms, and QOL in the current study are encouraging. With a favorable safety profile (with special reference to the safe nephrology profile, which is an emerging concern with many herbal products) as demonstrated in the study, Liv.52 emerges as a promising complementary or alternative therapeutic option for managing chronic liver diseases of mild to moderate severity. Although this trend is encouraging in terms of symptomatic improvement, improvement in liver parameters, and QOL in 12 weeks, larger duration studies with specific indications and endpoints based on therapeutic hepatology guidelines are warranted for further validation of these outcomes.

## Appendices

**Table 4:** Investigator, Site Details, Ethics committee and Approval date *Dropped **No enrollment Out of 40 total sites, 2 sites were dropped before site initiation. Further one site had no enrollment, hence there were only 37 active sites in the study. CTRI: Clinical Trials Registry-India

Site No.	Investigator Name	Site Name	Site Address	EC Name	Approval Number/ Details in CTRI	Approval Date	
							01	Dr. Atul V Rajkondawar	Seva Multispecialty Clinic	2nd Floor, Shrivardhan Complex, Panchsheel Sq., Near Big Bazar, Nagpur-10	Jasleen Hospital Institutional Ethics committee, Nagpur	No.: HWC/MSCD/PP/043/2021 https://ctri.nic.in/Clinicaltrials/WriteReadData/ethic/58143137DrAtul_ECApproval_Jasleenhospital.pdf	18-07-2022	
02	Dr. Yoshita Talmale	V G Memorial Hospital	256, New Ramdaspeth, Nagpur - 440012	Jasleen Hospital Institutional Ethics committee, Nagpur	No.: HWC/MSCD/PP/043/2021 https://ctri.nic.in/Clinicaltrials/WriteReadData/ethic/4562698697DrYoshita_ECApproval_Jasleenhospital.pdf	18-07-2022	
03	Dr. Ravindra Sawarkar	Vedanta Superspecialty Hospital	First Floor, Shree Radhey Heights Opposite Neeti-Gaurav Complex, Ramdaspeth, Nagpur, Maharashtra	Jasleen Hospital Institutional Ethics committee, Nagpur	No.: HWC/MSCD/PP/043/2021 https://ctri.nic.in/Clinicaltrials/WriteReadData/ethic/7975479252DrSawarkar_ECApproval_Jasleenhospital.pdf	18-07-2022	
04	Dr. Sumit Choudhary	Shri Krupa Hospital	Shop No.111 Ram Bhau Mhalgi Nagar, Taj Bagh, Mudkeshwar Road, Nagpur, Maharashtra 440034,	Jasleen Hospital Institutional Ethics committee, Nagpur	No.: HWC/MSCD/PP/043/2021 https://ctri.nic.in/Clinicaltrials/WriteReadData/ethic/1810069835ECApproval_DrSumeetchoudhary_Jasleenhospital.pdf	18-07-2022	
05	Dr. Shivnitwar Sachin Kishan	Lifepoint Multispeciality Hospital	145/1, Mumbai–Bangalore Highway, Near Hotel Sayaji, Bhumkar Chowk, Pune, Maharashtra 411057	LPR Ethics Committee, Pune	No.: HWC/MSCD/PP/043/2021 https://ctri.nic.in/Clinicaltrials/WriteReadData/ethic/6015674185HWC-MSCD-PP-043-2021_ECApproval-lifepoint.pdf	27-06-2022	
06	Dr. Vishwajeet Gaikwad	Imperial Multispecialty Hospital	Pingle Pride, Near Radha Swami Ashram, Chikhali, Pune - 411062	LPR Ethics Committee, Pune	No.: HWC/MSCD/PP/043/2021 https://ctri.nic.in/Clinicaltrials/WriteReadData/ethic/2541994469HWC-MSCD-PP-043-2021_EC	27-06-2022	
07*	Dr. Amol Lakshmanrao Dange	Skinovate Institute of Health & Research	Office NO 303 3 RD Floor, Royal Avenue S NO 18, Hissa NO 11/6, NR Hotel Shivar Garden Pimple Saudagar Rahatani, Pune - 411017	LPR Ethics Committee, Pune	No.: HWC/MSCD/PP/043/2021 https://ctri.nic.in/Clinicaltrials/WriteReadData/ethic/4725252894HWC-MSCD-PP-043-2021_ECApproval-Skinovate.pdf	27-06-2022	
08	Dr. Kanchan Saraf / Dr. Girish Bhatia	Medipoint Hospital Pvt. Ltd.	241/1, New D P Road, Aundh Baner Boundary, Baner Road, Pune, Maharashtra 411007	LPR Ethics Committee, Pune	No.: HWC/MSCD/PP/043/2021 https://ctri.nic.in/Clinicaltrials/WriteReadData/ethic/392341546HWC-MSCD-PP-043-2021_ECApproval-Medipoint.pdf	27-06-2022	
09	Dr. Aniket Oswal	Oswal Clinic	Shop No. 4, Nobel Residency, Bibwewadi, Kondhwa Road, Pune - 411037	LPR Ethics Committee, Pune	No.: HWC/MSCD/PP/043/2021 https://ctri.nic.in/Clinicaltrials/WriteReadData/ethic/730689633HWC-MSCD-PP-043-2021-ECApproval-OswalClinic.pdf	27-06-2022	
10	Dr. Ambana Gowda	Citizen Hospital	#14, 2nd Main, Disensary Road, Kalasipalya, Bangalore - 560002	Citizen Hospital Institutional Ethics Committee, Bangalore	No.: CH/EC/AP-02/30/06/2022 https://ctri.nic.in/Clinicaltrials/WriteReadData/ethic/479448210ECApproval_Citizenhospital.pdf	30-06-2022	
11	Dr. Fareeda Khannum	Healing Earth Multi speciality Ayurveda hospital	#149, Sector-5,Ring road,HSR layout, Bangalore	Citizen Hospital Institutional Ethics Committee, Bangalore	No.: CH/EC/AP-02/30/06/2022 https://ctri.nic.in/Clinicaltrials/WriteReadData/ethic/479448210ECApproval_Citizenhospital.pdf	30-06-2022	
12	Dr. Parth Patel	Hi-Tech Multispeciality Hospital	Sector - 3D, Plot no. 1180, Gandhinagar, Gujarat - 382003	Hitech Institutional Ethics Committee, Gandhinagar	No.: HWC/MSCD/PP/043/2021 https://ctri.nic.in/Clinicaltrials/WriteReadData/ethic/261626649DrParthPatel_ECApproval_hightechhospital.pdf	09-08-2022	
13	Dr. Jayesh Shah	Sharda Hospital	Sector -7, Gandhinagar, Gujarat	Hitech Institutional Ethics Committee, Gandhinagar	No.: HWC/MSCD/PP/043/2021 https://ctri.nic.in/Clinicaltrials/WriteReadData/ethic/7796888788DrJayesh_ECApprovalletter_hightechhospital.pdf	09-08-2022	
14	Dr. Sandeep Kumar Ojha	Jayyush Hospital	A-40, Mradul Park - 3, R.C., Technical Road, Chanakyapuri, Ghatlodiya, Ahmedabad	Hitech Institutional Ethics Committee, Gandhinagar	No.: HWC/MSCD/PP/043/2021 https://ctri.nic.in/Clinicaltrials/WriteReadData/ethic/7450367102DrSandeep_ECApproval_hightechhospital.pdf	09-08-2022	
15	Dr. Kuldeep Katariya	Jeevandan Hospital	OPD 2, Ground Floor, Bhelsangam Square, Danish Nagar, Bhopal - 462026	Institutional Ethics Committee Charak Hospital and Research Centre, Bhopal	No.: ECR/1562/Inst/MP/2021 https://ctri.nic.in/Clinicaltrials/WriteReadData/ethic/858235367ECApprovalLetter_DrKuldeepKatariya_Liv52study.pdf	26-08-2022	
16	Dr. Sanjay Ambhore	Shreya Clinic	4, Om Shiv Nagar, 60 Ft. Mandir Road, Lalghati, Bhopal, MP	Institutional Ethics Committee Charak Hospital and Research Centre, Bhopal	No.: ECR/1562/Inst/MP/2021 https://ctri.nic.in/Clinicaltrials/WriteReadData/ethic/9325887189ECApprovalLetter_DrSanjayAmbhore_Liv52study.pdf	26-08-2022	
17	Dr. Anil Sejwar	Balaji Children Hospital	75, Opp. Motia Talab Road, Post Office Bhopal, Kohefiza, Bhopal, Madhya Pradesh 462001	Institutional Ethics Committee Charak Hospital and Research Centre, Bhopal	No.: ECR/1562/Inst/MP/2021 https://ctri.nic.in/Clinicaltrials/WriteReadData/ethic/8531090187ECApprovalLetter_DrAnilSejwar_Liv52study.pdf	26-08-2022	
18	Dr. Navneet Arya	Sri Sai Institute of Ayurvedic Research and Medicine	Siaram (a Unit of Mansarovar Group of Institutions), Ward 84, Kolar Rd, Bhopal, Madhya Pradesh - 462042	Institutional Ethics Committee Charak Hospital and Research Centre, Bhopal	No: ECR/1562/Inst/MP/2021 https://ctri.nic.in/Clinicaltrials/WriteReadData/ethic/169626328ECApprovalLetter_DrNavneetArya_Liv52study.pdf	26-08-2022	
19	Dr. Brij Mohan	Brij Medical centre Pvt Ltd	94 E, Near Panki Police Station, Panki, Kanpur, UP	Institutional Ethics Committee Brij Medical Centre Pvt Ltd, Panki,Kanpur	No.: HWC/MSCD/PP/043/2021 https://ctri.nic.in/Clinicaltrials/WriteReadData/ethic/4268521008ECApproval_BrijMedicalcentrePvtLtd.pdf	27-09-2022	
20	Dr. Dilip Shah	St. Mother Teresa Multispecialty Hospital	Opp. Gram Panchayat Satpara Naka, Virar, Mumbai (West)	Vijay Vallabh Hospital Institutional Ethics Committee, Mumbai	No.: ECR/880/lnst/MH/201 7/R-R 2020 https://ctri.nic.in/Clinicaltrials/WriteReadData/ethic/805989835ECAPPROVAL_DRDILIPSHAH_Vijayvallabh.pdf	01-08-2022	
21	Dr. Rakesh Patil	Kkasturi Medicare Pvt. Ltd.	Harsh Niketan, Gaondevi Road, Behind Naurang Hotel, Bhayandar, Mumbai	Vijay Vallabh Hospital Institutional Ethics Committee, Mumbai	No.: ECR/880/lnst/MH/201 7/R-R 2020 https://ctri.nic.in/Clinicaltrials/WriteReadData/ethic/4538018091ECAPPROVAL_DrRakeshPatil_Vijayvallabh.pdf	01-08-2022	
22	Dr.Iswar Gilada	Unison Medicare & Research Centre	Unison Medicare, Maharukh Mansion, Alibhai Premji Marg, Grant Road East, Mumbai-400007, India	Vijay Vallabh Hospital Institutional Ethics Committee, Mumbai	No.: ECR/880/lnst/MH/2017/R-R 2020 https://ctri.nic.in/Clinicaltrials/WriteReadData/ethic/7508623964ECAPPROVAL_DRGILADA_Vijayvallabh.pdf	01-08-2022	
23	Dr. Aditya B Dixit	Apna Clinic	Shop NO 11, Block NO 7, ROW J, Rajiv Gandhi Nagar, Transit Camp, Mumbai, Maharashtra 400017	Vijay Vallabh Hospital Institutional Ethics Committee, Mumbai	No.: ECR/880/lnst/MH/201 7/R-R 2020 https://ctri.nic.in/Clinicaltrials/WriteReadData/ethic/6758817905ECAPPROVAL_DRADITYA_Vijayvallabh.pdf	01-08-2022	
24	Dr. Radha Gupta	Dr. Radha Gupta Clinic	Shop No. 46, A - wing, Vallabh CHS, 90 ft. Road, opp. Dharavi Police Station, Dharavi, Mumbai - 400017	Vijay Vallabh Hospital Institutional Ethics Committee, Mumbai	No.: ECR/880/lnst/MH/2017/R-R 2020 https://ctri.nic.in/Clinicaltrials/WriteReadData/ethic/1850875503ECAPPROVAL_DRRADHA_Vijayvallabh.pdf	01-08-2022	
25	Dr. Manish Kumar Singh	Vital Care Medical	2008, Awas Vikas & Panki Road., Kalyanpur, Kanpur, UP-208017	Institutional Ethics Committee Brij Medical Centre Pvt Ltd, Panki,Kanpur	No.: HWC/MSCD/PP/043/2021 https://ctri.nic.in/Clinicaltrials/WriteReadData/ethic/97539910ECApproval_VitalCareMedical.pdf	27-09-2022	
26	Dr. Sandeep Katiyar	Chest Care Clinic	112/369A, Swaroop Nagar, Kanpur	Institutional Ethics Committee Brij Medical Centre Pvt Ltd, Panki,Kanpur	No.: HWC/MSCD/PP/043/2021 https://ctri.nic.in/Clinicaltrials/WriteReadData/ethic/591947482ECApproval_ChestCareClinic.pdf	27-09-2022	
27	Dr. Pradeep Jain	Poddar Hospital	3510AB, Langer Key Balaji Ka Rasta, Near Nahargarh Road Police Station, Chandpole, Purani Basti, Jaipur, Rajasthan 302001	Institutional Ethics Committee Maharaja Agrasen Super Speciality Hospital, Jaipur, Rajasthan	No.: IEC/APV/OCT/2022/06 https://ctri.nic.in/Clinicaltrials/WriteReadData/ethic/6361692612DrPradeepJain_ECApproval.pdf	31-10-2022	
28	Dr. Mahesh Poddar	Medihub Multispecialty Clinic	269, Lane no. 4, Vashisht Marg, Raja Park, Jaipur, Rajasthan 302004	Institutional Ethics Committee Maharaja Agrasen Super Speciality Hospital, Jaipur, Rajasthan	No.: IEC/APV/OCT/2022/05 https://ctri.nic.in/Clinicaltrials/WriteReadData/ethic/288646263DrMahesh_ECApproval.pdf	31-10-2022	
29	Dr. Deepesh Thawani	Keemat Clinic	578, Sindhi Colony, Raja Park, Jaipur, Rajasthan	Institutional Ethics Committee Maharaja Agrasen Super Speciality Hospital, Jaipur, Rajasthan	No.: IEC/APV/OCT/2022/04 https://ctri.nic.in/Clinicaltrials/WriteReadData/ethic/4496561982DrDeepesh_ECApproval.pdf	31-10-2022	
30	Dr. P K Sharma	Maharaja Agrasen Multispecialty Hospital	84, Madan Gogar Marg, Vinoba Vihar, Model Town, Malviya Nagar. P.C. 302017, Jaipur, Rajasthan.	Institutional Ethics Committee Maharaja Agrasen Super Speciality Hospital, Jaipur, Rajasthan	No.: IEC/APV/OCT/2022/02 https://ctri.nic.in/Clinicaltrials/WriteReadData/ethic/6495713723DrPKSharma_ECApproval.pdf	31-10-2022	
31	Dr. Sameer Agarwal	Tulsi Hospital India Ltd	14/116-A. Civil Lines, Kanpur-208001	Institutional Ethics Committee Brij Medical Centre Pvt Ltd, Panki, Kanpur	No.: HWC/MSCD/PP/043/2021 https://ctri.nic.in/Clinicaltrials/WriteReadData/ethic/980273617ECApproval_TulsiHospitalIndiaLtd.pdf	27-09-2022	
32	Dr. Preeti Ahuja	Sukh Sahaj Clinic	118/72, Naseemabad Rd, Near Gumti Plaza, Gumti No.5, Kaushalpuri, Darshan Purwa, Kanpur, Uttar Pradesh 208012	Institutional Ethics Committee Brij Medical Centre Pvt Ltd, Panki,Kanpur	No.: HWC/MSCD/PP/043/2021 https://ctri.nic.in/Clinicaltrials/WriteReadData/ethic/8546910693ECApproval_SukhSahajClinic.pdf	27-09-2022	
33	Dr. Anil Bhargava	Charak Hospital & Research Centre	OPD 01,Ground Floor, Maharana Pratap Nagar, Jehangirabad, Bhopal - 462008	Institutional Ethics Committee Charak Hospital and Research Centre, Bhopal	No: ECR/1562/Inst/MP/2021 https://ctri.nic.in/Clinicaltrials/WriteReadData/ethic/4070154509ECApprovalLetterDrAnilBhargava_Liv52Study.pdf	15-09-2022	
34	Dr. Saurabh Chandra	Chandra Clinic	AK Marg, Adarsh Nagar, Jaipur, Rajasthan- 333023	Institutional Ethics Committee Maharaja Agrasen Super Speciality Hospital, Jaipur, Rajasthan	No.: IEC/APV/OCT/2022/07 https://ctri.nic.in/Clinicaltrials/WriteReadData/ethic/8669520836DrSaurabh_ECApproval.pdf	31-10-2022	
35**	Dr. Panchal Dharmendra	Diabetes Care and Hormone Clinic	5th Floor, Krishna Complex, Acher Cross Road, Opp. HP Petrol Pump, Sabarmati, Ahmedabad, Gujarat - 380005	Hitech Institutional Ethics Committee, Gandhinagar	No.: HWC/MSCD/PP/043/2021 https://ctri.nic.in/Clinicaltrials/WriteReadData/ethic/3630412851Liv-52_DrDharmendra_ECApprovalLetter.pdf	19-12-2022	
36	Dr. Sanjiv Gulati	Sarvottam Hospital	48 & 49 Gufa Mandir road, Janki Nagar, Lalghati, Bhopal 462030	Institutional Ethics Committee Charak Hospital and Research Centre, Bhopal	No.: ECR/1562/Inst/MP/2021 https://ctri.nic.in/Clinicaltrials/WriteReadData/ethic/448523983Approvalletter_Dr_SanjeevGulati.pdf	13-01-2023	
37*	Dr Shivakumar B.R	Dr. Ambedkar Medical College	Department of medicine, Dr Ambedkar medical college, Shampur main road, K. G Hally Bangalore	Citizen Hospital Institutional Ethics Committee, Bangalore	No.: CH/IEC/AP-01/04/02/23 https://ctri.nic.in/Clinicaltrials/WriteReadData/ethic/8752423302ECApproval_DrShivakumar.pdf	04-02-2023	
38	Dr. Anil Batra	Dr Anil Batra clinic	142, JK Road, Karamveer Nagar, Bharat Nagar, Indrapuri, Bhopal - 462022 Madhya Pradesh	Institutional Ethics Committee Charak Hospital and Research Centre, Bhopal	No: ECR/1562/Inst/MP/2021 https://ctri.nic.in/Clinicaltrials/WriteReadData/ethic/8710391690ECApproval_DrAnilBatra.pdf	13-02-2023	
39	Dr. Chandrashekhar Gilurkar	Gillurkar Multispeciality Hospital	20, Reshimbag umred road, Nagpur 440024	Jasleen Hospital Institutional Ethics committee, Nagpur	No.: HWC/MSCD/PP/043/2021 https://ctri.nic.in/Clinicaltrials/WriteReadData/ethic/853520502Jasleenhospital_Liv52_ECApproval.pdf	27-02-2023	
40	Dr. Akhil Sukhadeve	Surecare superspecialty Hospital	opp super speciality hospital ridge road, Vishvakarma nagar, tukdoji square nagpur - 440027	Jasleen Hospital Institutional Ethics committee, Nagpur	No.: HWC/MSCD/PP/043/2021 https://ctri.nic.in/Clinicaltrials/WriteReadData/ethic/1528314343DrAkhil_SurecareHospital_ECApproval.pdf	27-02-2023	

**Table 5:** Hepatic Laboratory Investigations (for Patients with Abnormal Values at Baseline) ALP: Alkaline phosphatase; ALD: Alcoholic liver disease; ALT: Alanine aminotransferase; AST: Aspartate aminotransferase; DIH: Drug-induced hepatotoxicity; EOS: End of study; LFT: Liver function test; NAFLD: Non-alcoholic fatty liver disease; NS: Not specified; SD: Standard deviation.

LFT Parameters	n	Baseline	EOS	Change from Baseline [Mean (SD)]	% Change from Baseline [Mean (SD)]	P-Value	Remains Abnormal, n (%)	Abnormal to Normal, n (%)	P-Value
Overall									
AST (U/L)	840	113.7 (98.20)	37.0 (39.30)	-76.7 (106.21)	-53.8 (45.23)	<0.0001	207 (24.64)	633 (75.36)	<0.0001
ALT (U/L)	729	123.8 (89.01)	36.9 (53.64)	-87.0 (103.44)	-61.1 (60.60)	<0.0001	137 (18.79)	592 (81.21)	<0.0001
Serum bilirubin (mg/dL)	347	2.0 (1.62)	0.7 (0.60)	-1.3 (1.71)	-8.3 (183.62)	<0.0001	103 (29.68)	244 (70.32)	<0.0001
ALP (U/L)	355	188.6 (120.54)	96.8 (54.41)	-91.8 (130.97)	-27.7 (100.03)	<0.0001	76 (21.41)	279 (78.59)	<0.0001
ALD									
AST (U/L)	320	112.5 (89.95)	36.9 (30.55)	-75.6 (96.85)	-52.9 (47.94)	<0.0001	74 (23.13)	246 (76.88)	<0.0001
ALT (U/L)	264	119.9 (92.90)	37.2 (36.32)	-82.7 (99.50)	-59.9 (38.67)	<0.0001	52 (19.70)	212 (80.30)	<0.0001
Serum bilirubin (mg/dL)	153	2.2 (1.63)	0.7 (0.52)	-1.5 (1.70)	-26.0 (131.11)	<0.0001	39 (25.49)	114 (74.51)	<0.0001
ALP (U/L)	123	178.8 (122.16)	86.0 (41.38)	-92.8 (127.64)	-27.9 (109.29)	<0.0001	19 (15.45)	104 (84.55)	<0.0001
NAFLD									
AST (U/L)	337	109.1 (90.99)	38.4 (51.19)	-70.7 (103.48)	-52.2 (46.54)	<0.0001	91 (27.00)	246 (73.00)	<0.0001
ALT (U/L)	299	122.2 (85.16)	40.1 (74.20)	-82.0 (113.49)	-58.0 (82.57)	<0.0001	55 (18.39)	244 (81.61)	<0.0001
Serum bilirubin (mg/dL)	125	1.6 (1.26)	0.7 (0.69)	-0.9 (1.42)	24.3 (238.93)	<0.0001	41 (32.80)	84 (67.20)	<0.0001
ALP (U/L)	165	193.7 (101.25)	107.0 (66.77)	-86.7 (119.94)	-29.0 (82.26)	<0.0001	42 (25.45)	123 (74.55)	<0.0001
DIH									
AST (U/L)	70	165.9 (177.26)	37.8 (27.46)	-128 (180.48)	-58.6 (29.43)	<0.0001	21 (30.00)	49 (70.00)	0.0004
ALT (U/L)	65	162.2 (112.98)	33.7 (31.19)	-129 (110.97)	-72.1 (27.43)	<0.0001	17 (26.15)	48 (73.85)	<0.0001
Serum bilirubin (mg/dL)	24	1.9 (1.41)	0.5 (0.30)	-1.4 (1.43)	-47.4 (57.18)	<0.0001	7 (29.17)	17 (70.83)	0.0206
ALP (U/L)	29	180.6 (38.47)	94.0 (35.77)	-86.6 (55.85)	-45.3 (23.56)	<0.0001	8 (27.59)	21 (72.41)	0.0079
Hepatitis									
AST (U/L)	113	98.5 (54.67)	32.5 (23.68)	-66.0 (61.04)	-58.1 (41.27)	<0.0001	21 (18.58)	92 (81.42)	<0.0001
ALT (U/L)	101	114.4 (64.02)	28.3 (18.42)	-86.2 (65.94)	-66.3 (43.18)	<0.0001	13 (12.87)	88 (87.13)	<0.0001
Serum bilirubin (mg/dL)	45	2.5 (2.26)	0.8 (0.72)	-1.8 (2.33)	-17.5 (195.24)	<0.0001	16 (35.56)	29 (64.44)	0.0263
ALP (U/L)	38	204.6 (206.16)	89.8 (32.14)	-115 (208.80)	-8.3 (159.09)	0.0017	7 (18.42)	31 (81.58)	<0.0001
Patients with diabetes and NAFLD									
AST (U/L)	10	76.2 (32.21)	37.6 (22.37)	-38.6 (46.55)	-38.3 (48.59)	<0.0277	4 (40.00)	6 (60.00)	NS
ALT (U/L)	12	91.7 (30.05)	46.6 (39.53)	-45.1 (49.42)	-46.5 (44.38)	<0.0091	5 (41.67)	7 (58.33)	NS
Serum bilirubin (mg/dL)	4	1.4 (0.09)	1.2 (0.96)	-0.2 (0.94)	-16.7 (67.59)	NS	1 (25.00)	3 (75.00)	NS
ALP (U/L)	4	143.0 (21.26)	126.3 (87.07)	-16.8 (71.04)	-15.3 (45.32)	NS	1 (25.00)	3 (75.00)	NS

**Table 6:** Mean (SD) CLDQ Scores for Patients (N=962) at Baseline and EOS (PP Analysis Set) Note: Chronic Liver Disease Questionnaire (CLDQ) scores ≥5 indicate "good", while <5 indicates "poor." CLDQ: Chronic Liver Disease Questionnaire; EOS: End of study; PP: Per protocol; QOL: Quality of life; SD: Standard deviation.

	Baseline	EOS	Change from Baseline	% Change from Baseline	P-value
Fatigued, Mean (SD)					
Fatigued (Tiredness)	4.7 (1.66)	6.4 (0.87)	1.7 (1.44)	56.5 (79.64)	0.005
Decreased strength	4.9 (1.55)	6.4 (0.80)	1.6 (1.40)	49.1 (65.51)	0.005
Decreased level of energy	4.9 (1.55)	6.5 (0.79)	1.5 (1.39)	46.1 (57.78)	0.005
Drowsiness (Feeling sleepy during the day)	5.3 (1.49)	6.6 (0.69)	1.3 (1.35)	37.8 (60.47)	0.005
Fatigued (Overall)	5.0 (1.43)	6.5 (0.70)	1.5 (1.25)	47.4 (56.02)	0.005
Emotional function, Mean (SD)					
Anxiety	5.8 (1.40)	6.7 (0.57)	0.9 (1.21)	25.9 (55.08)	0.005
Unhappiness	5.9 (1.24)	6.8 (0.54)	0.9 (1.10)	22.2 (41.13)	0.005
Irritability	5.9 (1.37)	6.7 (0.63)	0.8 (1.16)	22.9 (46.95)	0.005
Feeling depressed	6.0 (1.27)	6.8 (0.50)	0.8 (1.08)	19.3 (38.80)	0.005
Mood swings	6.1 (1.24)	6.8 (0.52)	0.7 (1.09)	18.5 (44.00)	0.005
Problems concentrating	6.0 (1.26)	6.7 (0.57)	0.7 (1.07)	18.6 (37.83)	0.005
Difficulty sleeping at night	5.9 (1.30)	6.7 (0.56)	0.8 (1.13)	21.6 (40.37)	0.005
Emotional function (Overall)	5.9 (1.11)	6.7 (0.45)	0.8 (0.94)	21.3 (35.85)	0.005
Abdominal symptoms, Mean (SD)					
Abdominal bloating	4.9 (1.61)	6.7 (0.64)	1.8 (1.51)	61.1 (91.88)	0.005
Abdominal pain/discomfort	4.7 (1.56)	6.7 (0.66)	2.0 (1.49)	66.6 (93.94)	0.005
Abdominal symptoms (Overall)	4.8 (1.49)	6.7 (0.60)	1.9 (1.43)	63.8 (88.39)	0.005
Worry, Mean (SD)					
Impact on family	6.1 (1.29)	6.9 (0.37)	0.7 (1.21)	23.1 (65.27)	0.005
Conditions getting worse	6.3 (1.22)	6.9 (0.31)	0.6 (1.16)	19.1 (60.70)	0.005
Worry (Overall)	6.2 (1.22)	6.9 (0.32)	0.7 (1.15)	21.1 (60.46)	0.005
Availability of liver activity, Mean (SD)					
Not able to eat as much as you like (Loss of Appetite)	5.1 (1.52)	6.7 (0.64)	1.6 (1.44)	48.9 (72.59)	0.005
Indigestion	5.4 (1.49)	6.7 (0.63)	1.3 (1.35)	38.6 (60.77)	0.005
Availability of liver activity (Overall)	5.2 (1.40)	6.7 (0.60)	1.5 (1.29)	43.8 (61.29)	0.005
Systemic symptoms, Mean (SD)					
Bodily pain	5.5 (1.46)	6.7 (0.63)	1.2 (1.30)	33.5 (57.86)	0.005
Shortness of breath	6.3 (1.11)	6.9 (0.42)	0.5 (0.97)	13.5 (37.49)	0.005
Muscle cramps	5.9 (1.47)	6.8 (0.51)	0.9 (1.34)	27.7 (56.41)	0.005
Dry mouth	6.2 (1.16)	6.8 (0.46)	0.7 (1.04)	16.6 (38.61)	0.005
Itching	6.5 (1.01)	6.9 (0.35)	0.4 (0.93)	11.9 (42.21)	0.005
Systemic symptoms (Overall)	6.1 (0.96)	6.8 (0.36)	0.7 (0.86)	20.7 (34.62)	0.005
QOL assessment (Overall), Mean (SD)	5.5 (0.94)	6.7 (0.39)	1.2 (0.84)	36.3 (39.53)	0.005
